# Traditional Chinese patent medicines for cancer treatment in China: a nationwide medical insurance data analysis

**DOI:** 10.18632/oncotarget.5711

**Published:** 2015-10-13

**Authors:** Min Wu, Peng Lu, Luwen Shi, Shao Li

**Affiliations:** ^1^ Department of Pharmacy Administration and Clinical Pharmacy, School of Pharmaceutical Sciences, Peking University, Beijing, China; ^2^ MOE Key Laboratory of Bioinformatics and Bioinformatics Division, TNLIST/Department of Automation, Tsinghua University, Beijing, China; ^3^ Institute of Automation, Chinese Academy of Sciences, Beijing, China

**Keywords:** traditional Chinese medicine, cancer treatment, medical insurance, nationwide survey, herb-drug network

## Abstract

Based on the nationwide survey into inpatients' utilization of the health service covered by China's urban basic medical insurance from 2008 to 2010, we analyzed the use rate, cancer profile and combined use of anticancer Chinese patent medicines (CPMs) on 51,382 insured cancer patients by using statistical, bi-clustering and network methods. We found that 42.4% of 51,382 cancer patients used 33 anticancer CPMs, and 51.7% used 71 anticancer Western medicines (WMs). The CPMs were most often used in lung (52%) and nasopharynx (52%) cancer patients, and least in bladder cancer (21%) and leukemia of unspecified cell type (21%) patients. The cost per patient for all 33 anticancer CPMs was 2069RMB, lower than that of the WMs (3458RMB). The cancer profile of commonly used CPMs and WMs for the top 17 cancers (>500 sampled patients) were provided, indicating anticancer CPMs had a broad spectrum of cancers and lacked selectivity in cancer treatment (CPM mean CV = 49%; WM mean CV = 152%). Moreover, 24.8% of the cancer patients used both CPMs and WMs, and CPM-WM combined use networks were constructed for four major cancers. This first nationwide analysis revealed the use characteristics and herb-drug combined use patterns of insurance covered anticancer CPMs in China. The study offers valuable information to guide future studies of the precision, safety and standard use of CPMs.

## INTRODUCTION

Chinese patent medicines (CPMs), as a new fashion to modernize traditional Chinese medicine (TCM), play an increasingly important role in China's medical practice. CPMs are produced by modern manufacturing processes in forms such as capsules and injections [1]. CPMs, together with Western medicines (WMs) and Chinese herbal medicines, are the three categories of drugs in China's National Basic Medical Insurance Drug Catalogue (NBMIDC) [2], since China launched the National Basic Medical Insurance System in 1998. Anticancer CPMs is a special category in NBMIDC anticancer drugs. Recently, several hospitals reported that anticancer CPMs are increasingly being used in cancer treatment, and even growing faster than Western drugs [3–5]. Moreover, TCM has showed bright prospects not only in the discovery of anticancer drugs [6], but also in the systematic therapy for complex diseases [7]. With the wide use and increasing interest of CPMs in cancer treatment, a comprehensive and in-depth analysis for anticancer CPMs is urgently required.

To meet such urgent needs, we analyzed the data from the first nationwide survey into inpatients' utilization of health services covered by China's Urban Basic Medical Insurance from 2008 to 2010. This investigation was conducted by the China Health Insurance Research Association affiliated with the Ministry of Human Resources and Social Security of China. The health insurance data can provide rich information to describe the clinical care and drug use in a large scale [8, 9]. Based on 51,382 insured cancer inpatients from the nationwide survey, we for the first time revealed the use characteristics as well as herb-drug combined use patterns in China, offering valuable information to improve the rational use of anticancer drugs in the future.

## RESULTS

### Overall use of anticancer CPMs and WMs in sampled cancer patients

**Figure 1:**
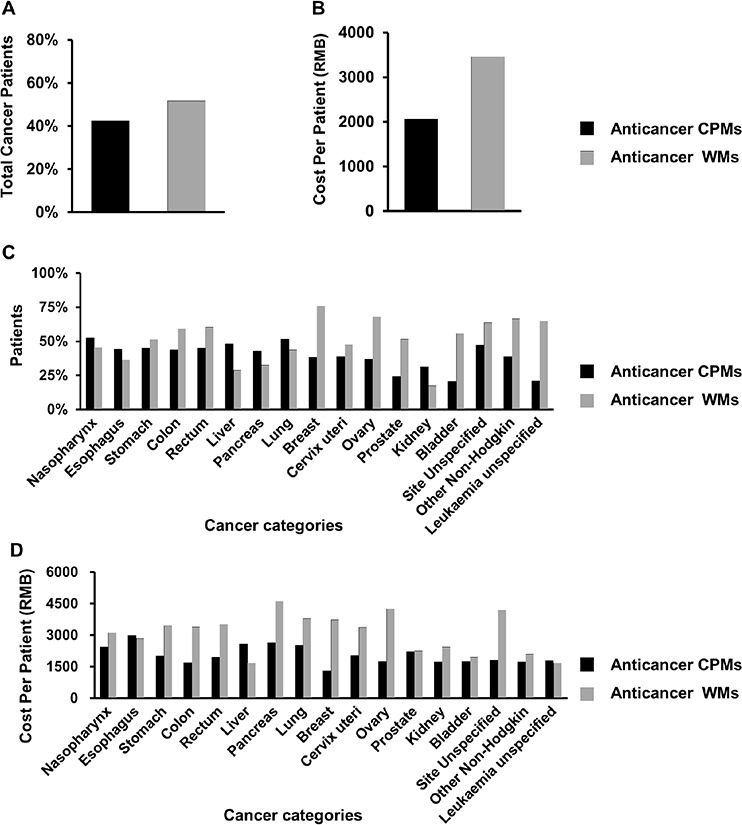

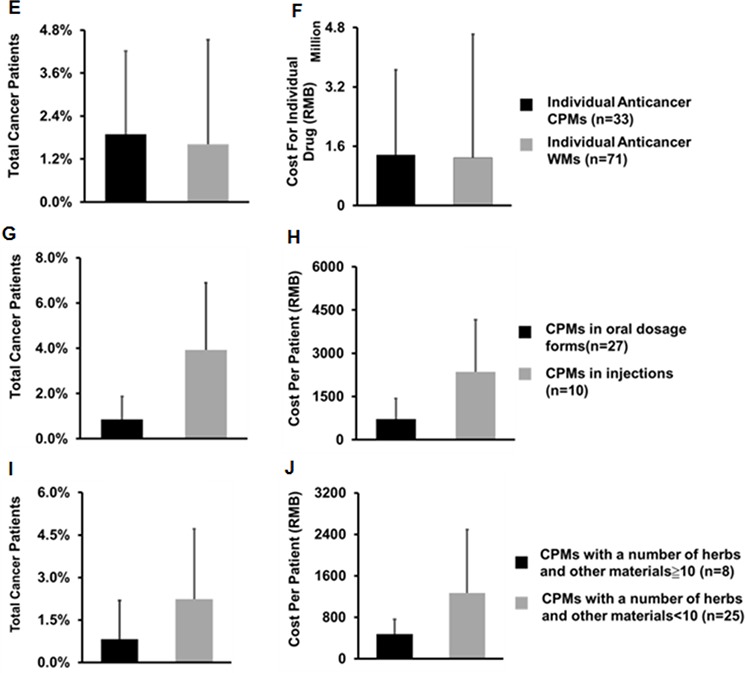
The overall use rate (A) and cost per patient (B) of 33 anticancer CPMs and 71 anticancer WMs, and their use rate (C) and cost per patient (D) in the top 17 cancers with a case number over 500. Use rate (E) and cost (F) of individual anticancer drugs in sampled cancer patients; use rate (G) and cost per patient (H) of anticancer CPMs in different formulations; use rate (I) and cost per patient (J) of anticancer CPMs with different numbers of raw materials

Of the 51,382 hospitalized cancer patients (Table S1), 67.9% (cost ratio 13.4%) used CPMs, 42.4% (cost ratio 9.1%; 2069 RMB cost per patient) used anticancer CPMs; 98.2% (cost ratio 86.4%) used WMs, and 51.7% (cost ratio 18.6%; 3458 RMB cost per patient) used anticancer WMs (Fig. 1A and 1B). Overall, 44,447 patients (87%) experienced 17 malignant cancers with each cancer having more than 500 cases (Table S2). 9 of the top 10 cancers are the same as those from a previously China national survey [10]. In top 17 cancers, use of 33 anticancer CPMs was highest in lung and nasopharynx cancer patients (both were 52%), and was higher than those using anticancer WMs (44% and 45%). Anticancer CPMs were also more commonly used than anticancer WMs for liver cancer (20% more), kidney cancer (14% more), pancreatic cancer (10% more), and esophagus cancer (8% more). By contrast, anticancer CPMs were used least for bladder cancer and leukemia of unspecified cell type (both were 21%) (Fig. 1C and 1D).

### Use of individual anticancer CPMs in sampled cancer patients

As shown in Fig. 1E–1J, the use rate (mean 1.9%) of individual anticancer CPMs in 51,382 cancer patients was significantly higher than that of individual anticancer WMs (mean 1.6%) (*P* = 0.017). There was no significant difference in the cost between individual anticancer CPMs (mean, 1.37 million RMB per drug) and that of individual anticancer WMs (mean, 1.29 million RMB per drug) (*P* = 0.069). Moreover, in subtypes of the anticancer CPMs, the use rate and cost of the CPMs injections or CPMs with less than 10 raw materials were significantly higher than those of oral formulations, or those with over 10 raw materials, respectively (all *P* < 0.05). The number of raw materials contained in an anticancer CPM was found to be significantly and negatively correlated with its usage (correlation coefficient = −0.449, *P* = 0.009; see data and diagram in Table S3).

### Raw materials and efficacy of commonly used anticancer CPMs

By calculating the total use frequency of 33 anticancer CPMs with the same materials, the 10 mostly used raw materials are Huang-Qi (*Astragali Radix*), Ren-Shen (*Ginseng Radix Et Rhizoma*), Ku-Shen (*Sophorae Flavescentis Radix*), Ban-Mao (*Mylabris*), Ci-Wu-Jia (*Acanthopanacis Senticosi Radix Et Rhizoma Seu Caulis*), Dang-Shen (*Codonopsis Pilosula*), Nv-Zhen-Zi (*Ligustri Lucidi Fructus*), Tu-Fu-Ling (*Smilacis Glabrae Rhizoma*), Ban-Zhi-Lian (*Scutellariae Barbatae Herba*), and Ya-Dan-Zi (*Bruceae Fructus*). The therapy effects of anticancer CPMs are mainly expressed as two types of traditional efficacy in their instructions. One is “eliminating pathogen” (*Qu-Xie* in Chinese) labeled as clearing away hot, relieving blood stasis, removing toxin, etc. for those Antitumor CPMs. For instance, all top 3 antitumor CPMs have an efficacy of clearing away hot. The other is “strengthening body resistance” (*Fu-Zheng* in Chinese) labeled as tonifying Qi, nourishing yin, etc. for those Adjuvant antitumor CPMs. Table 1 listed the individual efficacy of top 15 anticancer CPMs with use frequency more than 500 patients.

**Table 1:** Raw materials, labeled efficacy, and use frequency of top 15 anticancer CPMs used in cancer patients (>500 cases)

CPM name	NBMIDC subcategory	Raw material	Labeled efficacy	Use frequency
Shenqifuzheng Injection	Adjuvant antitumor CPM	*Astragali Radix, Codonopsis Pilosula*	Tonifying Qi and strengthening body resistance	4114
Fufangkushen Injection	Antitumor CPM	*Sophorae Flavescentis Radix, Smilacis Glabrae Rhizoma*	Clearing away hot, removing dampness, cooling blood, removing toxin, resolving hard mass, and relieving pain	3931
Aidi Injection	Antitumor CPM	Mylabris*, Ginseng Radix Et Rhizoma, Astragali Radix, Acanthopanacis Senticosi Radix Et Rhizoma Seu Caulis	Clearing away hot, removing toxin, resolving stagnation and dispersing masses	3761
Yadanziyou Injection (Oral emulsion, Soft capsule)	Antitumor CPM	Bruceae Fructus*	Antitumor; (Herb efficacy: Clearing away hot and removing toxin)	2683
Kangai Injection	Adjuvant antitumor CPM	*Astragali Radix, Ginseng Radix Et Rhizoma, Sophorae Flavescentis Radix*	Tonifying Qi and strengthening body resistance	2515
Fufangbanmao Capsule	Antitumor CPM	Mylabris*, Ginseng Radix Et Rhizoma, Astragali Radix, Acanthopanacis Senticosi Radix Et Rhizoma Seu Caulis, Sparganii Rhizoma, Scutellariae Barbatae Herba, Curcumae Rhizoma, Corni Fructus, Ligustri Lucidi Fructus, fel ursi, Glycyrrhizae Radix	Relieving blood stasis, resolving stagnation, removing toxin, and corroding sores	2098
Zhenqifuzheng Granule (Capsule, Tablet)	Adjuvant antitumor CPM	*Astragali Radix, Ligustri Lucidi Fructus*	Tonifying Qi and nourishing yin	1871
Huachansu Injection (Capsule, Tablet)	Antitumor CPM	Bufonis Corium*	Removing toxin, detumescence, and relieving pain	1387
Xiaoaiping Injection (Oral formulations)	Antitumor CPM	*Marsdenia tenacissima Caulis*	Antitumor, anti-inflammatory, and antiasthmatic	1339
Fufangzaofan Pill	Adjuvant antitumor CPM	*Melanteritum, Panacis Quinquefolii Radix, Hippocampus, Cinnamomi Cortex, Jujubae Fructus, Juglandis Semen*	Warming kidney, tonifying marrow, Qi, yin and blood, and stanching bleeding	1191
Huangqi Injection	Adjuvant antitumor CPM	*Astragali Radix*	Tonifying Qi and strengthening body resistance, pulse-invigorating and heart-nourishing, fortifying spleen and disinhibiting dampness	943
Pingxiao Capsule (Tablet)	Antitumor CPM	Curcumae Radix, Agrimoniae Herba, Trogopterori Faeces, Alumen, Nitrum, Toxicodendri Resina*, Aurantii Fructus, Strychni Semen Pulveratum*	Promoting blood circulation for removing blood stasis, relieving pain, resolving mass, clearing away hot, removing toxin, strengthening body resistance, and eliminating pathogen	762
Kanglaite Injection (Soft Capsule)	Antitumor CPM	*Jobstears Seed Oil*	Tonifying Qi, nourishing yin, and resolving mass	628
Fermental Preparation of Chongcaojun	Adjuvant antitumor CPM	*Cordyceps*	Supplementing lung and kidney, and tonifying essence Qi	624
Yixuesheng Capsule	Adjuvant antitumor CPM	*Asini Corii Colla, Testudinis Carapacis et Plastri Colla, Cervi Cornus Colla, Deer blood, Beef marrow, Hominis Placenta, Cervi Cornu Pantotrichum, Poria, Astragali Radix Praeparata Cum Melle, Paeoniae Radix Alba, Polygoni Multiflori Radix Praeparata, Jujubae Fructus, Crataegi Fructus, Hordei Fructus Germinatus, Galli Gigerii Endothelium Corneum, Anemarrhenae Rhizoma, Rhei Radix et Rhizom, Testa Arachidis*	Spleen invigorating and kidney nourishing, replenishing blood, supplying essences	548

* Toxic materials indicated in China's Pharmacopoeia or literature.

**Figure 2:**
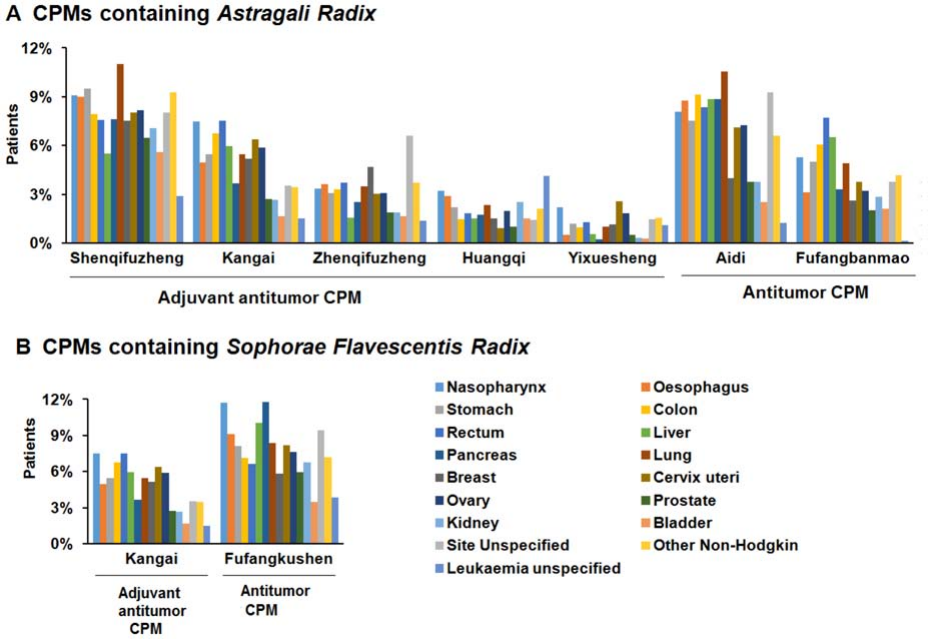
Use frequency of seven CPMs containing *Astragali Radix* (A) and two CPMs containing *Sophorae Flavescentis Radix* (B) that in both “antitumor” and “adjuvant antitumor” subcategories of CPM

We noticed that CPMs in two NBMIDC subcategories have overlapping raw materials. For example, in top 15 anticancer CPMs, five adjuvant antitumor CPMs and two antitumor CPMs (*Aidi* Injection and *Fufangbanmao* Capsule) has a herb material Huang-Qi (*Astragali Radix*). Both an antitumor CPM (*Fufangkushen* Injection) and an adjuvant antitumor CPM (*Kangai* Injection) contain the herb Ku-Shen (*Sophorae Flavescentis Radix*). These CPMs showed similar applicable indications (Fig. 2). Moreover, five antitumor CPMs including *Aidi* Injection and *Fufangbanmao* Capsule contain toxic materials indicated in China's Pharmacopoeia or literature (Table 1), which may reflect the TCM idea of “fighting poison with poison”.

### Cancer profile of anticancer CPMs and WMs by bi-clustering analysis

**Figure 3:**
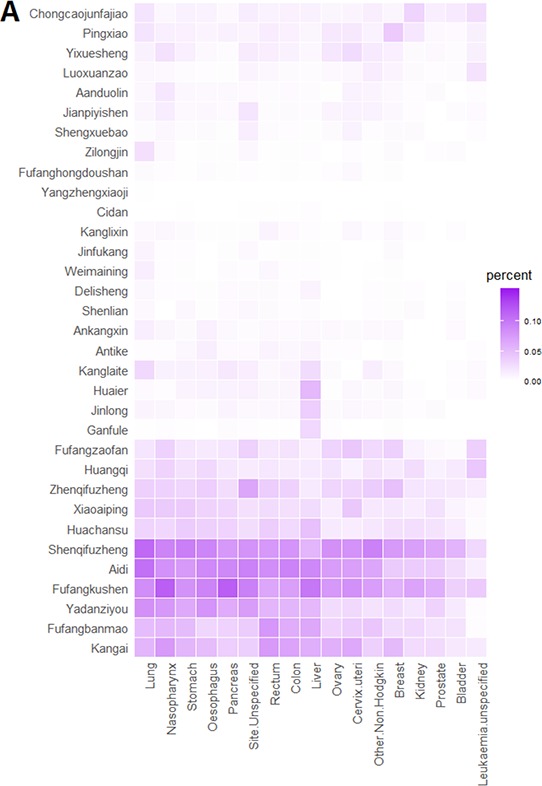

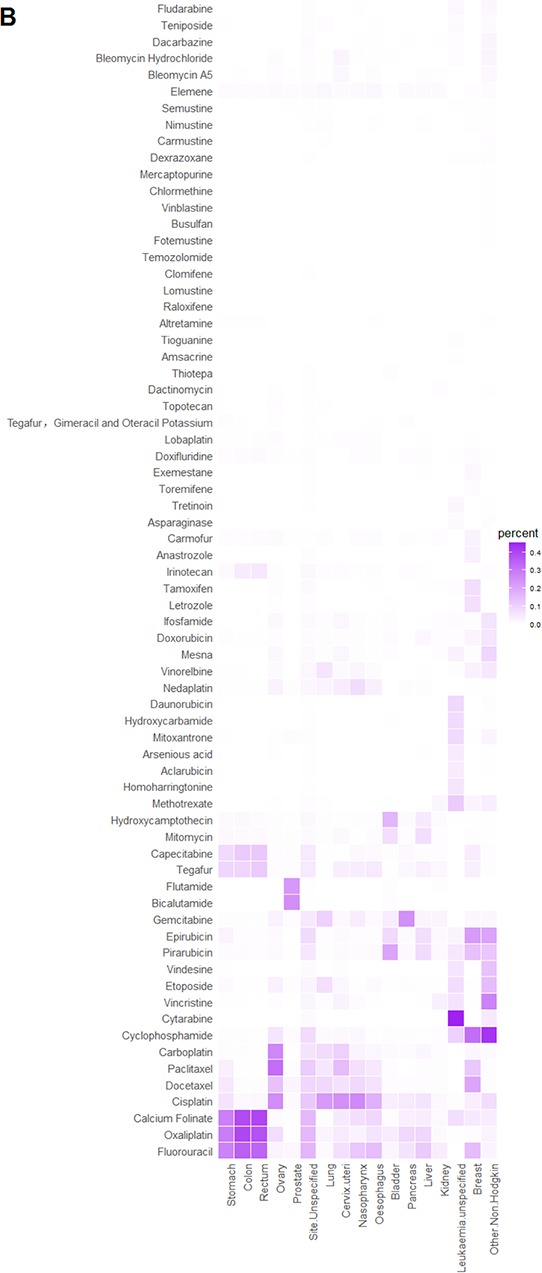
Cancer profiles of anticancer CPMs (A) and anticancer WMs (B)

Anticancer CPMs tended to be used for a broad spectrum of top 17 cancers (Fig. 3A). Few of these (*Huaier*, *Jinlong*, *Ganfule* and *Zilongjin*) showed selectivity but had a relatively low use rate (<6%) in the specific cancers. The top two frequently used anticancer CPMs (*Shenqifuzheng* injection and *Fufangkushen* injection) were used for almost all 17 types of cancers. By contrast, anticancer WMs were more specific in cancer therapy (Fig. 3B). Meanwhile, anticancer WMs for liver and kidney cancers were used at a lower rate than those for other cancers. Table S4 showed 22 anticancer WMs with use frequency more than 500 patients.

### Individual anticancer medicines for top 17 cancers

Individually, anticancer CPMs do not dominate the cancer therapy. The top use rate of individual anticancer CPMs in 17 cancers except liver and kidney cancers was much lower than that of anticancer WMs (Table 2). The highest use rate of the CPMs in 17 cancers was *Fufangkushen* injection in nasopharynx and pancreas cancers (12%), *a* value much lower than that of individual anticancer WMs (45% for Cytarabine in leukaemia unspecified). Furthermore, the most commonly used WM for stomach cancer was a regimen of Oxaliplatin/Fluorouracil/LV, whereas the NCCN guideline (Version 1.2008) recommended 5-FU, Capecitabine, ECF (Epirubicin/Cisplatin/Fluorouracil) or DCF (Docetaxel/Cisplatin/Fluorouracil), based on high-level evidence. The top two anticancer WMs commonly used for bladder cancer (Pirarubicin and Hydroxycamptothecin) in current data were absent in NCCN guideline (Version 1.2008).

**Table 2:** 17 cancers (>500 patients) and the top used anticancer medicines

Cancer	Top 3 commonly used anticancer CPMs (use rate, %)			Top 3 commonly used anticancer WMs (use rate, %)		
Nasopharynx	Fufangkushen (12%)	Shenqifuzheng (9.1%)	Aidi (8.1%)	Cisplatin (26%)	Fluorouracil (12%)	Docetaxel (8.7%)
Oesophagus	Fufangkushen (9.1%)	Shenqifuzheng (9.0%)	Aidi (8.8%)	Cisplatin (18%)	Fluorouracil (15%)	Calcium Folinate (8.9%)
Stomach	Shenqifuzheng (9.5%)	Fufangkushen (8.1%)	Aidi (7.5%)	Oxaliplatin (29%)	Calcium Folinate (28%)	Fluorouracil (27%)
Colon	Aidi (9.1%)	Shenqifuzheng (7.9%)	Fufangkushen (7.1%)	Oxaliplatin (39%)	Calcium Folinate (39%)	Fluorouracil (35%)
Rectum	Aidi (8.4%)	Fufangbanmao (7.7%)	Shenqifuzheng (7.6%)	Calcium Folinate (40%)	Oxaliplatin (37%)	Fluorouracil (33%)
Liver	Fufangkushen (10%)	Aidi (8.8%)	Fufangbanmao (6.5%)	Fluorouracil (12%)	Oxaliplatin (8.9%)	Pirarubicin (7.6%)
Pancreas	Fufangkushen (12%)	Aidi (8.9%)	Shenqifuzheng (7.6%)	Gemcitabine (25%)	Oxaliplatin (8.7%)	Fluorouracil (6.1%)
Lung	Shenqifuzheng (11%)	Aidi (11%)	Fufangkushen (8.4%)	Cisplatin (22%)	Gemcitabine (10%)	Docetaxel (8.7%)
Breast	Shenqifuzheng (7.5%)	Fufangkushen (5.8%)	Kangai (5.2%)	Cyclophosphamide (32%)	Epirubicin (22%)	Docetaxel (20%)
Cervix uteri	Fufangkushen (8.2%)	Shenqifuzheng (8.0%)	Aidi (7.1%)	Cisplatin (24%)	Paclitaxel (15%)	Carboplatin (11%)
Ovary	Shenqifuzheng (8.2%)	Fufangkushen (7.6%)	Aidi (7.3%)	Paclitaxel (32%)	Carboplatin (27%)	Cisplatin (25%)
Prostate	Shenqifuzheng (6.5%)	Fufangkushen (6.0%)	Aidi (3.7%)	Bicalutamide (25%)	Flutamide (23%)	Docetaxel (22%)
Kidney	Shenqifuzheng (7.1%)	Fufangkushen (6.8%)	Aidi (3.8%)	Fluorouracil (4.1%)	Vincristine (3.5%)	Gemcitabine (2.5%)
Bladder	Shenqifuzheng (5.6%)	Fufangkushen (3.5%)	Aidi (2.5%)	Pirarubicin (20%)	Hydroxycamptothecin (16%)	Epirubicin (8.2%)
Site Unspecified	Fufangkushen (9.4%)	Aidi (9.0%)	Shenqifuzheng (8.0%)	Fluorouracil (16%)	Oxaliplatin (16%)	Calcium Folinate (16%)
Other Non-Hodgkin	Shenqifuzheng (9.3%)	Fufangkushen (7.2%)	Aidi (6.6%)	Cyclophosphamide (43%)	Vincristine (28%)	Epirubicin (20%)
Leukaemia unspecified	Huangqi (4.1%)	Fufangkushen (3.9%)	Fufangzaofan (3.4%)	Cytarabine (45%)	Methotrexate (12%)	Cyclophosphamide (10%)

Table 3 illustrates the mean and maximum use rate of anticancer CPMs and WMs in top 17 cancers, respectively. The coefficient of the variation (CV) value, characterizing the extent of variability of the use rate of anticancer CPMs, ranged from 24% to 93%, with a mean of 49%. This result was significantly lower than that of anticancer WMs, which ranged from 97% to 255%, with a mean of 152%. The results once again indicated less selectivity of anticancer CPMs in cancer therapy.

**Table 3:** Use rate of individual anticancer medicine in 17 cancers (> 500 patients)

Drugs	Category	Maximum rate (cancer)	Use rate in 17 cancers		
			Mean	CV	Mean of CV
Shenqifuzheng	CPM	11.0% (Lung)	7.7%	24%	49%
Fufangkushen	CPM	11.8% (Pancreas)	7.7%	30%	
Aidi	CPM	10.6% (Lung)	6.8%	40%	
Kangai	CPM	7.5% (Rectum)	4.7%	40%	
Yadanziyou	CPM	8.2% (Lung)	4.5%	57%	
Fufangbanmao	CPM	7.7% (Rectum)	3.9%	48%	
Zhenqifuzheng	CPM	6.6% (Site Unspecified)	3.1%	42%	
Huachansu	CPM	4.7% (Liver)	2.5%	43%	
Xiaoaiping	CPM	4.1% (Cervix uteri)	2.5%	46%	
Fufangzaofan	CPM	4.1% (Cervix uteri)	2.2%	51%	
Huangqi	CPM	4.1% (Leukaemia unspecified)	2.0%	41%	
Pingxiao	CPM	3.9% (Breast)	1.3%	61%	
Kanglaite	CPM	2.8% (Lung)	0.9%	93%	
Chongcaojunfajiao	CPM	3.1% (Kidney)	1.2%	57%	
Yixuesheng	CPM	2.6% (Cervix uteri)	1.1%	61%	
Fluorouracil	WM	34.9% (Colon)	11.4%	99%	152%
Oxaliplatin	WM	39.2% (Colon)	10.0%	127%	
Calcium Folinate	WM	39.6% (Rectum)	10.0%	129%	
Cisplatin	WM	26.2% (Nasopharynx)	9.8%	97%	
Cyclophosphamide	WM	42.6% (Other Non-Hodgkin)	6.2%	196%	
Docetaxel	WM	20.2% (Breast)	4.9%	118%	
Paclitaxel	WM	31.9% (Ovary)	5.5%	152%	
Epirubicin	WM	22.2% (Breast)	4.6%	149%	
Pirarubicin	WM	20.0% (Bladder)	4.3%	136%	
Carboplatin	WM	26.5% (Ovary)	3.7%	176%	
Gemcitabine	WM	24.6% (Pancreas)	3.7%	159%	
Tegafur	WM	11.7% (Rectum)	3.5%	103%	
Capecitabine	WM	12.2% (Rectum)	2.8%	144%	
Etoposide	WM	14.7% (Other Non-Hodgkin)	2.3%	168%	
Vinorelbine	WM	5.8% (Lung)	1.3%	144%	
Vincristine	WM	27.6% (Other Non-Hodgkin)	2.6%	255%	
Nedaplatin	WM	7.5% (Nasopharynx)	1.4%	150%	
Mitomycin	WM	7.4% (Bladder)	1.4%	158%	
Hydroxycamptothecin	WM	16.2% (Bladder)	1.7%	221%	
Irinotecan	WM	5.0% (Rectum)	0.9%	169%	

### Combined use networks of anticancer CPMs and WMs

**Figure 4:**
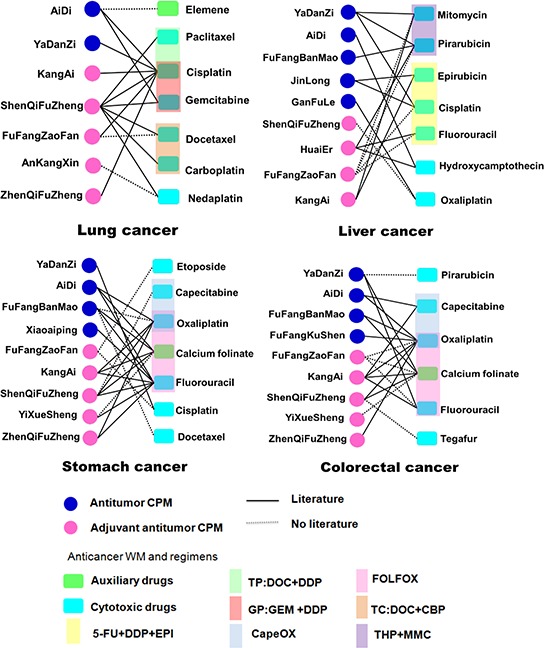
Combined use network of anticancer CPMs and WMs in four major cancers The combined links in networks marked with the full lines were supported by clinical evidence (Table S5), and those with the dotted lines lacked clinical reports.

In this survey, a total of 12,743 (24.8%) patients used both anticancer CPMs and WMs simultaneously during their hospital stays. To obtain the combined use patterns of both medicines, a CPM-WM combined use network for four major cancers in China was constructed (Fig. 4). Among the high frequent combinations, adjuvant antitumor CPMs were more commonly used in conjunction with WMs in treating lung cancer. The gastrointestinal cancers, in particular stomach and colorectal cancers, shared similar combination profiles, in which CPMs tended to band together with the first line WM regimens, such as FOLFOX4. Meanwhile, adjuvant antitumor CPMs including *Shenqifuzheng* injection and antitumor CPMs including *Aidi* injection were involved in all four cancers together with WMs. Parts of these CPM-WM combinations were supported by clinical evidence as reported (Table S5).

## DISCUSSION

This nationwide analysis characterizes how CPMs are used to fight cancer in China. In general, anticancer CPMs have gained increased popularity and been used in almost half (42.4%) of investigated cancer patients. The cost per patient for all anticancer CPMs is lower than that of anticancer WMs. We found that CPMs with less raw materials are relatively more popular in treating cancers (Fig. 1; Table S3). This is partly due to CPM injections that usually composed of less raw materials are used more commonly than other CPMs. And the less raw materials may make the CPMs easier to understand and more acceptable for clinicians. Compared with 71 anticancer WMs, the use rate of 33 anticancer CPMs was higher than in liver, kidney, pancreatic, and esophagus cancers. There are two probable reasons. Firstly, these four cancers may have less specific and effective anticancer WMs than other cancers, leading to a lower use rate of anticancer WMs. Secondly, some first line drugs for the liver cancer and kidney cancer such as Sorafenib and Sunitinib were not covered by NBMIDC, making the use rate of anticancer WMs much lower in liver cancer (29%) and kidney cancer (17%). By contrast, the anticancer CPMs are widely used in various cancers, and thus showing a relatively higher rate in these four cancers especially in liver and kidney cancers.

In this study, a “widespread and dispersive” charateristic with low selectivity for the use of anticancer CPMs has been revealed (Table 2, Table 3 and Fig. 3). Although the mean usage of anticancer CPMs was significantly higher than their western counterparts, the use rates of top anticancer CPMs (no more than 12%) were much lower than those of top WMs (a maximum of 45%) in 17 main cancers except kidney cancer (Table 2). The results suggest the individual anticancer CPMs are not dominant in a specific cancer treatment. One possible reason is that CPMs always regulate overall health of human body rather than simply fight against a specific cancer. A typical case is *Shenqifuzheng* injection, the most often used adjuvant antitumor CPM. This injection, broadly used for almost all 17 cancers, is composed of Huang-Qi (*Astragali Radix*) and Dang-Shen (*Codonopsis Pilosula*). Both of the two herbs contain immunomodulatory ingredients, and can improve the immune function of cancer patients receiving chemotherapy [11–14]. Thus, *Shenqifuzheng* injection could be widely used as an efficacious adjuvant to prevent immunity impairment that usually occurs in therapy of various cancers. Another reason for the low selectivity of CPMs is that the precise efficacy of CPMs is not quite clear in clinical use. Anticancer CPMs mainly labeled their traditional efficacy (Table 1). However, the applicable cancer type was not clearly stated in instructions or just indicated as “et al.” or “advanced cancers” for many CPMs. Thus, the specific and evidence-based indications are still required to improve the rational use of CPMs. This study also indicates the subcategory classification of CPMs in NBMIDC needs to be further clarified, since both CPM subcategories have similar clinical use patterns and overlapped herb materials such as *Astragali Radix* (an immunomodulatory herb [11,12]) and *Sophorae Flavescentis Radix* (an anti-tumor herb [15,16]) (Fig. 2). Special safety caution should also be paid to CPM injections and CPMs containing toxic ingredients.

The CPMs and WMs are commonly used in combination and work in concert for cancer treatment. Most of the herb-drug pairs identified by our network analysis (Fig. 4) have clinical evidence (Table S5), often reported as improving quality of life of patients and reducing adverse reactions of chemotherapy. Thus, CPMs may offer potential therapeutic benefits for cancer patients by boosting efficiency and decreasing toxicity caused by WMs. Other herb-drug pairs lacking of references deserve further investigation. More solid evidence and whether new safety risks can be generated from the herb-drug interactions needs further studies. A standard program will be of great significance to guide the combined use of CPMs and WMs.

Although our data only catch sampled patients in China and the results need to be validated in larger samples, this analysis for the first time addressed the clinical use of anticancer CPMs in a China nationwide. This study provides valuable information to improve the standardization, rationalization and internationalization of the clinical use of Chinese medicines.

## MATERIALS AND METHODS

### National-wide sampling

Data was from a nationwide survey for inpatients' utilization of health services covered by China's urban basic medical insurance from 2008 to 2010. All patients were firstly sorted by discharge time and then randomly sampled from China's 29 provincial-level administrative regions. The inpatients came from 2,576 hospitals in 22 provinces, 3 autonomous regions, and 4 centrally-administered municipalities. The percentage of the sampled inpatients who had medical insurance coverage was 2% in the centrally-administered municipalities and provincial capitals, 5% in the prefecture-level cities, and 10% in the counties.

### Drugs and cancer patients' data

The drugs, medication records and cost for 51,382 malignant tumor inpatients, including 11209 cases (21.8%) in 2008, 16850 cases (32.8%) in 2009, and 23323 cases (45.4%) in 2010, were retrieved from the sampling data. The data on two types of drugs were collected, including CPMs (registered and approved by China State Food and Drug Administration) and WMs (all modern medicines including saline, glucose and other auxiliary drugs).

### NBMIDC recorded anticancer CPMs and WMs

A total of NBMIDC-covered 33 anticancer CPMs (calculated by the number of generic names after different dosage forms were merged) and 71 anticancer WMs (calculated by the number of generic names of the active pharmaceutical ingredients) were used in sampled cancer patients. For anticancer CPMs, 17 fall into the NBMIDC “Antitumor” subcategory and 16 into the “Adjuvant antitumor” subcategory. The number of raw materials (herbs, animals or others) contained in each anticancer CPMs ranges from 1 to 22, and 124 materials get involved. Anticancer WMs include 56 cytotoxic, 9 hormone, 4 auxiliary and 2 other drugs.

### Statistical, bi-clustering, and network analysis

Use-frequency of a drug (CPM or WM) refers to the total number of patients in which a drug was used in the medication records. Use rate of a drug was the percentage of the total cancer patients or patients with a specific cancer who used the drug. Cost ratio means Ratio of the total drug cost. A Mann-Whitney rank sum test was used to evaluate the statistical significance of the use rate and cost between the anticancer CPMs and anticancer WMs, as well as a comparison analysis for different types of anticancer CPMs. A Spearman rank correlation analysis was used to analyze the correlation between the rank of use rates and the rank of number of raw materials contained in each anticancer CPMs.

Bi-clustering method was used to analyze the cancer profile of anticancer CPMs and WMs. A bipartite graph was first created for drugs and various types of cancers, in which nodes denoted drugs or cancers, and edges denoted the use of a drug for a particular cancer. The weights of edges were expressed as the use rate of drug, which represented the percentage of patients with a certain cancer who used drug. Then, hierarchical clustering was performed for drugs using all of the row vectors to cluster together similar drugs. Similarly, hierarchical clustering was performed for the various types of cancers to cluster together similar cancers. The order of drugs and cancers was adjusted according to the results of the clustering and a heat map was generated.

A network approach was used to analyze the combined use of CPMs and WMs [17]. The network nodes represented drugs, and the edges represented the combined relationship between two drugs. The combined relationship was measured by two indicators. One was the frequency of the combined use, which referred to the number of cancer patients who used drugs in combination. The other was the mutual information entropy [17]. The effective combined relationships were screened (total number of combined use >50 times, or total combined mutual information >0.005) for constructing CPM-WM combined use networks (see Supplementary text).

## SUPPLEMENTARY DATA AND TABLES






