# Supplementary material for: Traditional Chinese patent medicines for cancer treatment in China: a nationwide medical insurance data analysis
Source: Oncotarget. 2015 Oct 13;6(35):38283–95. doi: 10.18632/oncotarget.5711 (PMC4741999; doi:10.18632/oncotarget.5711)
Supplement: Supplementary file 1 [file oncotarget-06-38283-s001.pdf]

SUPPLEMENTARY DATA

Bi-clustering analysis for cancer profile of anticancer drugs

Bi-clustering was used to analyze the cancer profile of the anticancer CPMs and WMs. Here, a bipartite graph was first created for the drugs and various types of cancers. In this graph, the nodes denoted the drugs or cancers, and the edges denoted the use of a drug for a particular cancer. The weights of the edges were expressed as the use rate of the drug, which represented the percentage of patients with a certain cancer who used the drug. The calculation of the formula is as follows:

$$p(d,t) = \frac{f(d,t)}{f(t)}$$

Where d is the drug; t is the tumor; $p(d, t)$ is the use rate of the drug d; $f(t)$ is the number of patients with tumor t; and $f(d, t)$ is the number of patients with tumor t in which drug d is used.

An adjacency matrix for the bipartite graph was then created, according to the relationship between the drugs and the cancers:

$$\begin{array}{c} \\ drug_1 \\ drug_2 \\ drug_3 \end{array} \begin{array}{c} \begin{array}{cc} icd_1 & icd_2 \end{array} \\ \begin{bmatrix} x_{11} & x_{12} \\ x_{21} & x_{22} \\ x_{31} & x_{32} \end{bmatrix} \end{array}$$

The vertical axis represents the drugs, and the horizontal axis represents the cancer categories. A drug corresponds to a row vector, while a cancer corresponds to a column vector. Then, hierarchical clustering was performed for the drugs using all of the row vectors to cluster together similar drugs. Similarly, hierarchical clustering was performed for the various types of cancers to cluster together similar cancers. The order of the drugs and cancers was adjusted according to the results of the clustering. A heat map was generated based on this matrix, where the relevant drugs and cancers were clustered into modules.

Construction of a CPM-WM combined use network by mutual information analysis

A combined use network was established for the CPMs and Western drugs. The nodes of this network represented the drugs, and the edges represented the combined relationship between the two drugs. The combined relationship was mainly measured by two indicators. One indicator was the frequency of the combined use, which referred to the number of cancer patients who used drugs in combination. The other indicator was the mutual information entropy, which was calculated by the following formula [12]:

$$\mathrm{mie}(X,Y) = p(x,y) \log \frac{p(x,y)}{p(x)p(y)}$$

Where P (x, y) is the rate of the combined use of drugs X and Y, which is equal to the frequency of the combined use, divided by the number of all the cancer patients; P (x) is the use rate of drug X, which is equal to its use frequency, divided by the number of all the cancer patients; and P (y) refers to the use rate of drug Y. The effective combined relationships were screened (total number of combined use > 50 times, or total combined mutual information > 0.005) for the purpose of constructing a CPM-WM combined use network.

Supplementary Table S1: Characteristics of the 51,382 cancer patients

Age	Cases	Percentage
0–5	95	0.2%
6–9	88	0.2%
10–15	115	0.2%
16–19	115	0.2%
20–29	654	1.3%
30–39	2445	4.8%
40–49	7466	14.5%
50–59	14309	27.8%
60–69	12668	24.7%
70–79	10585	20.6%
80-	2604	5.1%
Unknown	238	0.5%
**Gender**	**Cases**	**Percentage**
Female	24033	46.8%
Male	27311	53.2%
Unknown	38	0.1%

Supplementary Table S2: The ICD-10 codes, categories, and frequency of the sampled cancers

	ICD-10	Cancer categories	Abbreviations*	Frequency
1	C34	Malignant neoplasm of bronchus and lung	Lung	9285
2	C80	Malignant neoplasm without specification of site	Site Unspecified	6741
3	C50	Malignant neoplasm of breast	Breast	5902
4	C16	Malignant neoplasm of stomach	Stomach	4478
5	C18	Malignant neoplasm of colon	Colon	3208
6	C22	Malignant neoplasm of liver and intrahepatic bile ducts	Liver	2942
7	C20	Malignant neoplasm of rectum	Rectum	2756
8	C15	Malignant neoplasm of oesophagus	Oesophagus	1601
9	C56	Malignant neoplasm of ovary	Ovary	1383
10	C85	Other and unspecified types of non-Hodgkin lymphoma	Other Non-Hodgkin	1355
11	C25	Malignant neoplasm of pancreas	Pancreas	808
12	C95	Leukaemia of unspecified cell type	Leukaemia unspecified	737
13	C67	Malignant neoplasm of bladder	Bladder	728
14	C11	Malignant neoplasm of nasopharynx	Nasopharynx	706
15	C53	Malignant neoplasm of cervix uteri	Cervix uteri	680
16	C64	Malignant neoplasm of kidney, except renal pelvis	Kidney	647
17	C61	Malignant neoplasm of prostate	Prostate	599
18	C73	Malignant neoplasm of thyroid gland		483
19	C78	Secondary malignant neoplasm of respiratory and digestive organs		437
20	C54	Malignant neoplasm of corpus uteri		432
21	C90	Multiple myeloma and malignant plasma cell neoplasms		431
22	C79	Secondary malignant neoplasm of other and unspecified sites		326
23	C71	Malignant neoplasm of brain		305
24	C92	Myeloid leukaemia		293
25	C91	Lymphoid leukaemia		260
26	C32	Malignant neoplasm of larynx		258
27	C24	Malignant neoplasm of other and unspecified parts of biliary tract		217
28	C23	Malignant neoplasm of gallbladder		213
29	C00	Malignant neoplasm of lip		193

(*Continued*)

	ICD-10	Cancer categories	Abbreviations*	Frequency
30	C55	Malignant neoplasm of uterus, part unspecified		193
31	C76	Malignant neoplasm of other and ill-defined sites		192
32	C44	Other malignant neoplasms of skin		188
33	C41	Malignant neoplasm of bone and articular cartilage of other and unspecified sites		180
34	C17	Malignant neoplasm of small intestine		143
35	C77	Secondary and unspecified malignant neoplasm of lymph nodes		124
36	C26	Malignant neoplasm of other and ill-defined digestive organs		111
37	C43	Malignant melanoma of skin		107
38	C02	Malignant neoplasm of other and unspecified parts of tongue		98
39	C49	Malignant neoplasm of other connective and soft tissue		94
40	C81	Hodgkin lymphoma		80
41	C38	Malignant neoplasm of heart, mediastinum and pleura		79
42	C48	Malignant neoplasm of retroperitoneum and peritoneum		72
43	C21	Malignant neoplasm of anus and anal canal		65
44	C07	Malignant neoplasm of parotid gland		60
45	C66	Malignant neoplasm of ureter		60
46	C93	Monocytic leukaemia		60
47	C37	Malignant neoplasm of thymus		58
48	C63	Malignant neoplasm of other and unspecified male genital organs		57
49	C45	Mesothelioma		53
50	C57	Malignant neoplasm of other and unspecified female genital organs		52
51	C82	Follicular lymphoma		45
52	C03	Malignant neoplasm of gum		43
53	C40	Malignant neoplasm of bone and articular cartilage of limbs		42
54	C14	Malignant neoplasm of other and ill-defined sites in the lip, oral cavity and pharynx		41
55	C62	Malignant neoplasm of testis		38

(*Continued*)

	ICD-10	Cancer categories	Abbreviations*	Frequency
56	C65	Malignant neoplasm of renal pelvis		38
57	C30	Malignant neoplasm of nasal cavity and middle ear		36
58	C31	Malignant neoplasm of accessory sinuses		33
59	C94	Other leukaemias of specified cell type		32
60	C74	Malignant neoplasm of adrenal gland		30
61	C13	Malignant neoplasm of hypopharynx		29
62	C19	Malignant neoplasm of rectosigmoid junction		29
63	C96	Other and unspecified malignant neoplasms of lymphoid, haematopoietic and related tissue		29
64	C83	Non-follicular lymphoma		28
65	C08	Malignant neoplasm of other and unspecified major salivary glands		26
66	C04	Malignant neoplasm of floor of mouth		25
67	C05	Malignant neoplasm of palate		25
68	C06	Malignant neoplasm of other and unspecified parts of mouth		24
69	C09	Malignant neoplasm of tonsil		24
70	C60	Malignant neoplasm of penis		23
71	C68	Malignant neoplasm of other and unspecified urinary organs		22
72	C72	Malignant neoplasm of spinal cord, cranial nerves and other parts of central nervous system		22
73	C84	Mature T/NK-cell lymphomas		21
74	C51	Malignant neoplasm of vulva		18
75	C10	Malignant neoplasm of oropharynx		17
76	C75	Malignant neoplasm of other endocrine glands and related structures		16
77	C52	Malignant neoplasm of vagina		14
78	C88	Malignant immunoproliferative diseases		13
79	C69	Malignant neoplasm of eye and adnexa		12
80	C01	Malignant neoplasm of base of tongue		11
81	C70	Malignant neoplasm of meninges		11
82	C33	Malignant neoplasm of trachea		10
83	C58	Malignant neoplasm of placenta		9

(*Continued*)

	ICD-10	Cancer categories	Abbreviations*	Frequency
84	C39	Malignant neoplasm of other and ill-defined sites in the respiratory system and intrathoracic organs		5
85	C47	Malignant neoplasm of peripheral nerves and autonomic nervous system		5
86	C12	Malignant neoplasm of piriform sinus		3
87	C97	Malignant neoplasms of independent (primary) multiple sites		2
88	C46	Kaposi sarcoma		1

*Abbreviations are only given for the top 17 cancers with more than 500 patients

Supplementary Table S3: Correlation between the number of raw materials and the use rate of anticancer CPM.

Supplementary Table S4: Anticancer Western medicines used in the malignant tumor patients (>500 patients)

	Drug	Category	NBMIDC Subcategory	Use Frequency
1	Fluorouracil	WM	Cytotoxic drugs	6518
2	Oxaliplatin	WM	Cytotoxic drugs	5768
3	Calcium Folinate	WM	Auxiliary drugs	5695
4	Cisplatin	WM	Cytotoxic drugs	5415
5	Cyclophosphamide	WM	Cytotoxic drugs	3599
6	Docetaxel	WM	Cytotoxic drugs	3421
7	Paclitaxel	WM	Cytotoxic drugs	3026
8	Epirubicin	WM	Cytotoxic drugs	2826
9	Pirarubicin	WM	Cytotoxic drugs	2138
10	Carboplatin	WM	Cytotoxic drugs	2022
11	Gemcitabine	WM	Cytotoxic drugs	1957
12	Tegafur	WM	Cytotoxic drugs	1883
13	Capecitabine	WM	Cytotoxic drugs	1783
14	Etoposide	WM	Cytotoxic drugs	1353
15	Vinorelbine	WM	Cytotoxic drugs	1022
16	Vincristine	WM	Cytotoxic drugs	948
17	Cytarabine	WM	Cytotoxic drugs	704
18	Nedaplatin	WM	Cytotoxic drugs	616
19	Mitomycin	WM	Cytotoxic drugs	582
20	Hydroxycamptothecin	WM	Cytotoxic drugs	567
21	Irinotecan	WM	Cytotoxic drugs	516
22	Tamoxifen	WM	Hormone medicines	515

Supplementary Table S5: Clinical evidence for CPM-WM combinations.
